# A Case Report of Hairy Cell Leukemia Presenting Concomitantly with Sweet Syndrome

**DOI:** 10.1155/2014/823286

**Published:** 2014-02-18

**Authors:** Mohammad Alkayem, Waina Cheng

**Affiliations:** ^1^Internal Medicine Department, Lincoln Medical and Mental Health Center, 234 E 149th Street, Bronx, NY 10451, USA; ^2^Hematology and Oncology Department, Lincoln Medical and Mental Health Center, 234 E 149th Street, Bronx, NY 10451, USA

## Abstract

Hairy cell leukemia and Sweet syndrome are both uncommon hematological diagnoses. We present a patient who was admitted with fevers, pancytopenia, pneumonia, and rash. Diagnostic bone marrow biopsy demonstrates Hairy cell Leukemia and skin biopsy demonstrates neutrophils infiltration consistent with Sweet syndrome. The patient was treated with purine analogs with resolution of the cytopenias, infection, and rash.

## 1. Introduction

Hairy cell leukemia is an uncommon lymphoproliferative disorder often presenting with cytopenias, infections, and splenomegaly. In this case report, we will discuss a presentation of hairy cell leukemia with Sweet syndrome.

## 2. Case Report

A-52-year-old male presented to Lincoln Medical and Mental Health Center in March 2012 with fever, chills, dyspnea, and productive cough for 3 days. The physical exam was significant for tachycardia, tachypnea, fever of 102°F, and fine crackles heard in the right side of the chest. The patient's CBC showed neutropenia with ANC 900, mild anemia with hemoglobin 12.2 g/dL, and thrombocytopenia with platelets 79 × 10^3^. Initial blood smear demonstrated a limited number of white blood cells. A chest X-ray revealed right lower lobe consolidation. Computerized tomography (CT) scan of chest and abdomen revealed enlargement of the mediastinal lymph nodes and a mildly enlarged spleen. Within 24 hours of admission, the patient went into respiratory failure requiring ventilator support. He was subsequently placed on antibiotics for community acquired pneumonia with improvement in symptoms. On the 3rd day of hospitalization, physical exam was notable for development of a generalized erythematous papular rash on the abdomen and vesicles and bullae on the extremities. A punch skin biopsy was performed and the patient was started on systemic steroids with improvement of the rash.

**Figure 1:**
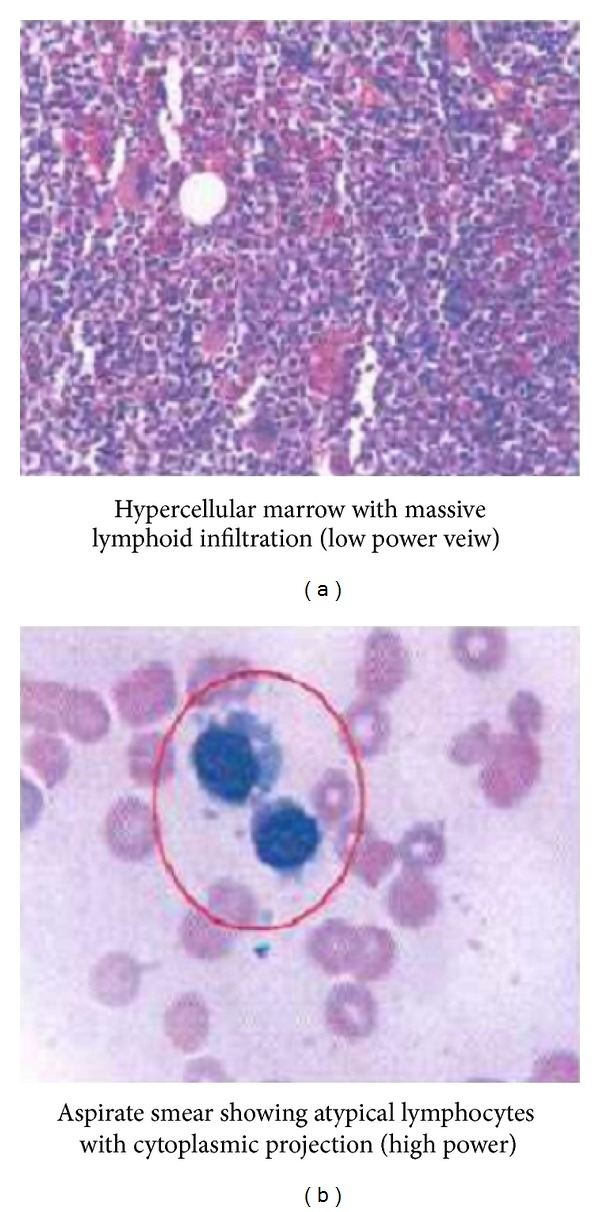
Bone marrow biopsy showed infiltration with hairy cells.

**Figure 2:**
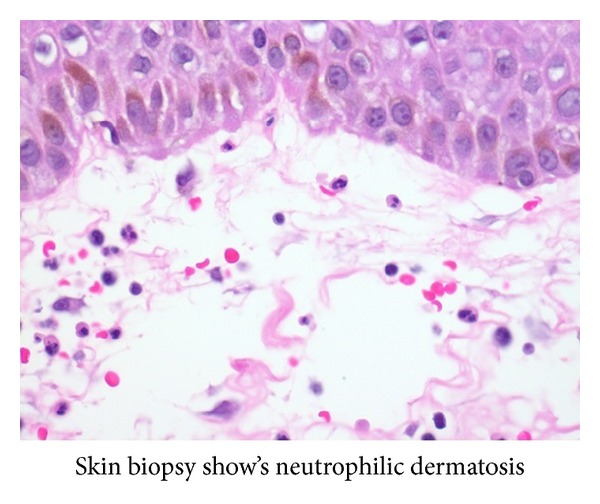
Infiltration with neutrophils (Sweet syndrome).

One week later, despite clinical improvement, patient had persistent pancytopenia. A diagnostic bone marrow biopsy was performed. No aspirate was able to be obtained despite multiple attempts. Bone marrow core biopsy show large lymphocytes with small cytoplasmic projections (Figure 1). The neoplastic cells stained positive for CD45, CD19, CD20, CD11c, CD22, CD25, and CD103, consistent with hairy cell leukemia. Concurrently, the skin biopsy showed neutrophilic dermatosis, consistent with Sweet syndrome (Figure 2).

Given resolution of his pneumonia, the patient received cladribine 0.1 mg/kg intravenous continuous infusion daily for 7 days as inpatient. The use of neupogen was deferred in the setting of neutrophilic dermatosis with concerns of possibly exacerbating the rash. The rash resolved after administration of chemotherapy. The patient was placed on prophylactic antibiotics, antivirals, and antifungals during the duration of myelosuppression. Within three months, his ANCED recovered and prophylactic medications were stopped. In six months, his CBC normalized with Hg of 13 g/dL and platelet 263 × 10^3^. Followup bone marrow biopsy showed persistent involvement by hairy cell leukemia. Since the patient remained asymptomatic, undomiciled, and unwilling, treatment for residual disease was deferred.

## 3. Discussion

Hairy cell leukemia is an uncommon B cell lymphoproliferative disorder which was first described in 1958 by Bouroncle et al. [1]. It represents 2% of all leukemia and 1% of all lymphomas. The pathogenesis is unknown. Clinically, the patient often presents with early satiety secondary to splenomegaly, fatigue and weakness secondary to anemia, bleeding secondary to thrombocytopenia, or life threatening infection secondary to granulocytopenia. However, patients can be asymptomatic and be diagnosed incidentally in the setting of cytopenias. A blood smear will show large lymphocytes with abundant cytoplasm with small cytoplasmic projections. A bone marrow aspirate is often not obtainable due to diffuse fibrosis. Core biopsy will show infiltration of characteristic hairy cells. Immunophenotyping via flow cytometry will show surface antigens CD20, CD25, CD103, and CD11c [2]. First line treatment is with a purine analog, which this patient received.

Sweet syndrome, also known as acute febrile neutrophilic dermatosis, is first described by Dr. Sweet in 1964 [3]. He characterized a syndrome of abrupt fevers, peripheral leukocytosis, and erythematous painful skin lesions. The dermatologic manifestations are secondary to dermal infiltrate of neutrophils. Diagnosis is based on the presence of 2 major and 4 minor criteria, listed below (Table 1). Extracutaneous manifestations involving the eyes, joints, lungs, and kidneys have also been described. Sweet syndrome can be subdivided into 3 categories depending on their etiology, classical, malignancy associated, and drug induced. Within malignancy associated Sweet syndrome, acute myelogenous leukemia is the most common etiology [4, 5]. Sweet syndrome associated with hairy cell leukemia is rare. On, the literature review, there have only been nine cases reported [6–10]. Of interest in this particular patient is the manifestation of Sweets syndrome in the setting of neutropenia not neutrophilia. This makes treatment of the underlying hairy cell leukemia difficult since it is unclear whether neupogen injection to increase neutrophil count will exacerbate the skin lesions.

## 4. Conclusion

This is an interesting case of a patient with hairy cell leukemia who presents with pancytopenia and Sweet syndrome. It is important for the oncologist to consider underlying malignancy especially hematologic ones in patients who present with fever and skin lesions.

## Figures and Tables

**Table 1:** The diagnostic criteria for sweet syndrome.

Major criteria	(i) Abrupt onset of painful erythematous plaques or nodules
	(ii) Histopathologic evidence of a dense neutrophilic infiltrate without evidence of leukocytoclastic vasculitis

Minor criteria	(i) Pyrexia > 38°C
	(ii) Association with underlying hematologic or visceral malignancy, inflammatory disease, pregnancy, or infection
	(iii) Response to treatment with systemic glucocorticoids
	(iv) Abnormal laboratory values at presentation

## References

[1] Bouroncle BA, Wiseman BK, Doan CA (1958). Leukemic reticuloendotheliosis. *Blood*.

[2] Grever MR, Lozanski G (2011). Modern strategies for hairy cell leukemia. *Journal of Clinical Oncology*.

[3] Sweet RD (1964). An acute febrile neutrophilic dermatosis. *The British Journal of Dermatology*.

[4] Paydas S (2013). Sweet’s syndrome: a revisit for hematologist and oncologists. *Critical Reviews in Oncology/Hematology*.

[5] Cohen PR (2007). Sweet’s syndrome—a comprehensive review of an acute febrile neutrophilic dermatosis. *Orphanet Journal of Rare Diseases*.

[6] Ventura F, Rocha J, Pereira T, Marques H, Pardal F, Brito C (2009). Sweet syndrome as the presenting symptom of hairy cell leukemia. *Dermatology Online Journal*.

[7] Levy RM, Junkins-Hopkins JM, Turchi JJ, James WD (2002). Sweet syndrome as the presenting symptom of relapsed hairy cell leukemia. *Archives of Dermatology*.

[8] Chang S, Chau W, Liu M, Ho C (1999). Acute febrile neutrophilic dermatosis (Sweet’s syndrome) in hairy cell leukemia: a case report. *Zhonghua Yi Xue Za Zhi*.

[9] Dalrì P, Boi S, Cristofolini M (1982). Sweet syndrome: presenting symptom of hairy cell leukemia with fatal infection by *Pneumocystis carinii*. *Haematologica*.

[10] Kramers C, Raemaekers JMM, van Baar HMJ, de Pauw BE, Horrevorts AM (1992). Sweet’s syndrome as the presenting symptom of hairy cell leukemia with concomitant infection by *Mycobacterium kansasii*. *Annals of Hematology*.

